# Increased misophonia in self-reported Autonomous Sensory Meridian Response

**DOI:** 10.7717/peerj.5351

**Published:** 2018-08-06

**Authors:** Agnieszka B. Janik McErlean, Michael J. Banissy

**Affiliations:** 1 Goldsmiths, University of London, London, United Kingdom; 2 Bath Spa University, Bath, United Kingdom

**Keywords:** ASMR, Misophonia, Synaesthesia, Sensation, Sound

## Abstract

**Background:**

Autonomous Sensory Meridian Response (ASMR) is a sensory experience elicited by auditory and visual triggers, which so far received little attention from the scientific community. This self-reported phenomenon is described as a relaxing tingling sensation, which typically originates on scalp and spreads through a person’s body. Recently it has been suggested that ASMR shares common characteristics with another underreported condition known as misophonia, where sounds trigger negative physiological, emotional and behavioural responses. The purpose of this study was to elucidate whether ASMR is associated with heightened levels of misophonia.

**Methods:**

The Misophonia Questionnaire (MQ) was administered to individuals reporting to experience ASMR and to age and gender matched controls.

**Results:**

Compared to controls ASMR group scored higher on all subscales of MQ including the Misophonia Symptom Scale, the Misophonia Emotions and Behaviors Scale and the Misophonia Severity Scale.

**Discussion:**

Individuals reporting ASMR experience have elevated levels of misophonia.

## Introduction

Sensitivity to sound, especially sound produced by humans, is termed misophonia, which literally means ‘hatred of sound’ (Jastreboff & Jastreboff, 2002). Although misophonia is not formally recognized as a disorder, recent evidence suggests that clinical symptomatology of misophonia resembles that of obsessive compulsive disorder (OCD; Schröder, Vulink & Denys, 2013; Johnson et al., 2013; Schwartz, Leyendecker & Conlon, 2011; Wu et al., 2014). Indeed, an association between OCD and misophonia has been recently reported (Wu et al., 2014; Zhou, Wu & Storch, 2017). Common features include aversive reactions such as heightened anxiety to specific triggers, avoidance or the need to complete compulsions. People who suffer from misophonia often find sounds such as eating, coughing or breathing extremely distressing. Such triggers often induce anger and anxiety as well as a strong autonomic arousal (Kumar et al., 2017). There are large individual differences in terms of the severity of the symptoms ranging from minimal discomfort to extreme responses such as violent outbursts or self-harming (Rouw & Erfanian, 2017). In more severe cases individuals who suffer from misophonia may avoid situations where such triggers could be encountered which, in turn, may interfere with day-to-day functioning (Cavanna & Seri, 2015; Wu et al., 2014; Edelstein et al., 2013).

Interestingly, anecdotal reports and recent findings suggest that there may be an increased incidence of misophonia among individuals who experience Autonomous Sensory Meridian Response (ASMR) (Janik McErlean & Banissy, 2017; Fredborg, Clark & Smith, 2017; Barratt, Spence & Davis, 2017; Barratt & Davis, 2015). ASMR is a self-reported sensory phenomenon described by those capable to experience it as a tingling sensation, which originates on scalp and spreads down the spine to the limbs. This experience is elicited by certain auditory and visual triggers including whisper, crisp sounds and personal attention (Janik McErlean & Banissy, 2017; Fredborg, Clark & Smith, 2017; Barratt & Davis, 2015). ASMR is reported to be highly relaxing and enjoyable, drawing hundreds of thousands of people every day to YouTube channels dedicated to this phenomenon. For instance, GentleWhispering, which is one of the most popular channels producing ASMR content has over one million subscribers and over 470,000,000 views (as of June, 2018).

While ASMR sensations are described as pleasurable and relaxing (Barratt & Davis, 2015; Janik McErlean & Banissy, 2017), many viewers of ASMR channels find certain sounds included in the videos distressing. This suggests that ASMR experience is a heterogenous phenomenon and that triggers commonly used in ASMR videos may result in vastly different reactions. These anecdotal reports have been corroborated by recent findings. For instance, although whispering is commonly reported to trigger pleasant tingling sensations it has also been reported as unpleasant in some circumstances (Janik McErlean & Banissy, 2017; Fredborg, Clark & Smith, 2017; Barratt & Davis, 2015). Interestingly, as much as 25.3% of the ASMR-responders in Janik McErlean & Banissy’s study (2017) reported “eating sounds”, which are common misophonia inducers (Wu et al., 2014; Zhou, Wu & Storch, 2017), to be unpleasant and uncomfortable. This trigger received by far the most negative evaluation of all listed triggers including amongst others finger tapping, crinkly plastic and typing. This is in line with recent findings which showed that “chewing sounds” were among one of the least likely triggers to induce ASMR experience (Fredborg, Clark & Smith, 2017). Interestingly, Rouw & Erfanian (2017) recently reported that a large portion of their misophonic participants (49%) also reported experiencing pleasurable tingling sensations induced by different sounds further suggesting a potential overlap between the two phenomena. At the same time, Barratt, Spence & Davis (2017) found that 43% of ASMR self-reporters stated that they experience misophonia when provided with a description of the phenomenon.

Furthermore, it has been suggested that misophonia and ASMR might represent two ends of the same spectrum of sound sensitivity and that they may be linked to synaesthesia, which has been reported to be more common in ASMR-responders compared to general population (Barratt & Davis, 2015). All these phenomena appear to share at least one similar characteristic—namely, they all rely on the inducer–concurrent relationship. In ASMR human generated sounds induce pleasurable tingling sensation while a negative physical and affective reaction is found in misophonia (Wu et al., 2014; Zhou, Wu & Storch, 2017; Edelstein et al., 2013).

The aim of this study was to establish whether ASMR is associated with heightened levels of misophonia. To do so the Misphonia Questionnaire (Wu et al., 2014), which consists of three subscales—the Misophonia Symptom Scale (MSS), the Misophonia Emotions and Behaviors Scale (MEBS), and the Misophonia Severity Scale—was administered to a group of individuals reporting ASMR experiences and to age and gender matched controls.

## Materials & Methods

This study was conducted online and was approved by the ethical committee of Goldsmiths University of London. Participants were first presented with information about the study. They were also informed that the study is anonymous and that their participation is voluntary and that they may withdraw at any point without any consequences. Participants were requested to give electronic consent prior to commencing the study.

### Participants

The sample included in the primary analysis included sixty four ASMR—responders (40 female, 24 male; age *M* = 28.81, SD = 6.05, range: 18–38) and sixty eight controls (52 female, 16 male; age *M* = 26.75, SD = 11.20, range: 18–66). The two groups did not significantly differ in terms of age [*t*(104.395) =  − 1.326, *p* = .188] or gender [*χ*^2(1,*N*=132)^ = 3.047, *p* = .081). The ASMR sample was recruited via a Facebook site dedicated to ASMR (https://www.facebook.com/groups/ASMRGroup/). The following description of ASMR was provided: “ASMR can be defined as a pleasurable tingling sensation which originates on scalp and spreads down the spine and through the whole body and which is typically induced by certain sounds (e.g., turning pages, crinkly wrapping paper, finger tapping), watching someone perform repetitive mundane actions (e.g., folding towels, going through items in a handbag), watching someone closely inspecting day-to-day objects, hearing whisper, watching someone’s hair being brushed or watching videos with various role plays (visit to a doctor, spa or a shop)”. All ASMR-responders confirmed they experience ASMR based on this description. Participants recruited from the ASMR Facebook site who indicated they did not experience ASMR were excluded from the analysis. Similarly, in order to avoid any potential misunderstanding and false positive answers participants recruited among university students and acquaintances who reported to experience ASMR were also excluded from the primary analysis. Control participants were recruited among acquaintances and student population and were either given credit points, cash vouchers, or were entered into a prize draw. Participants were included in the control group based on a negative answer to a question whether they experience ASMR, which was accompanied by the same aforementioned description of the phenomenon.

### Measures

The Misphonia Questionnaire (Wu et al., 2014) consists of three parts: the Misophonia Symptom Scale, the Misophonia Emotions and Behaviors Scale and the Misophonia Severity Scale. Misophonia Symptom Scale (MSS) establishes whether a person experiences any sound sensitivities by asking participants to rate how much each statement describes them on a 0 to 4 scale (0—*not at all true*, 4—*always true)*. This scale includes seven items such as e.g., “people eating”, “repetitive tapping” and “rustling”. A score of 14 or higher on this scale means that, on average, a participant scored greater than or equal to a score of "2" ("sometimes") for all questions and thus is treated as a cut-off score suggesting a presence of misophonia as per Wu and colleagues (2014). An additional open-ended question was included where participants could add and rate another sound not listed in the questionnaire. This question was not included in the computation of the final scores as many answers fell under the categories already included in the questionnaire.

The Misophonia Emotions and Behaviors Scale (MEBS) refers to emotional and behavioural responses elicited by misophonia. It asks the participants to indicate how often their sound sensitivities lead to outcomes such as e.g., “leave the environment to a place where the sound(s) cannot be heard anymore”, “actively avoid certain situations in anticipation of the sound” or “have violent thoughts“ on a 0 to 4 scale with 0 being “Never” and 4 being “Always.” This scale contains ten items. An additional open-ended question was included where participants could write another response not listed in the questionnaire. This question was not included in the computation of the final scores as many answers fell under the categories already included in the questionnaire. Misophonia Symptom Scale and Misophonia Emotions and Behaviors Scale are summed to form the Misophonia Questionnaire Total score (MQ Total), ranging from 0 to 68.

The Misophonia Severity Scale is based on the NIMH Global Obsessive-Compulsive Scale (NIMH GOCS; Murphy, Pickar & Alterman, 1982) which was modified to reflect the misophonia symptoms (Wu et al., 2014). Participants are asked to rate the severity of their sound sensitivity by choosing one of fifteen possible ratings (1—minimal, 15—very severe.) A score greater than or equal to 7 indicates ‘moderate sound sensitivity’ which interferes with daily life and thus is treated as a cut-off for clinically significant symptoms. Participants were asked to tick 0 if they had no sound sensitivity. This study was conducted online.

## Results

**Figure 1:**
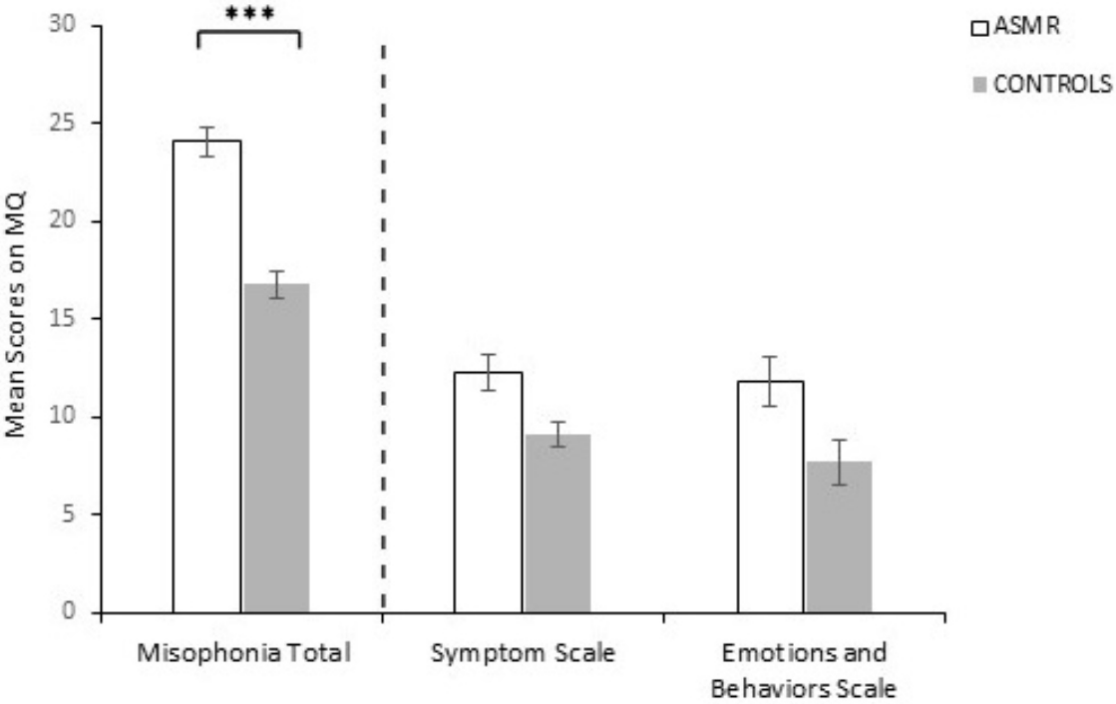
Mean responses for ASMR-responders (*N* = 64) and controls (*N* = 68) on the Misophonia Questionnaire (Total, Symptom, Emotions and behaviors scale). Error bars represent SEM. ^∗∗∗^*p* < 0.001.

Scores on the Misophonia Questionnaire Total (MQ Total, Cronbach’s alpha = .872) were analysed using an independent samples *t*-test which revealed a significant group difference [*t*(130) = 4.185, *p* ≤ .001, Cohen’s *d* = 0.73) with ASMR-responders scoring higher (*M* = 24.09, *SD* = 10.48) than controls (*M* = 16.80, *SD* = 9.51; Fig. 1). To further elucidate whether this result was due to group differences on either the Misophonia Symptom Scale (Cronbach’s alpha = .829), or the Misophonia Emotions and Behaviors Scale (Cronbach’s alpha = .874) a 2 (group: controls, ASMR-responders) ×2 (subscales: MSS, MEBS) ANOVA was conducted. This revealed no interaction (*F*(1, 130) = .603, *p* = .439, ŋ*p*^2^ = .005) and a main effect of group [*F*(1, 130) = 17.513, *p* ≤ .001, ŋ*p*_2_ = .119] with ASMR-responders scoring overall higher on both subscales (*M* = 12.05, *SE* = .62) compared to controls (*M* = 8.40, *SE* = .60). In addition, 36% of ASMR-responders compared to 22% non-responders scored 14 and above on the Misophonia Symptom Scale, which is treated as clinically significant misophonia symptoms. However, this difference in proportions between the two groups was statistically non-significant [*χ*^2(1,*N*=132)^ = 1.74, *p* = .186).

**Figure 2:**
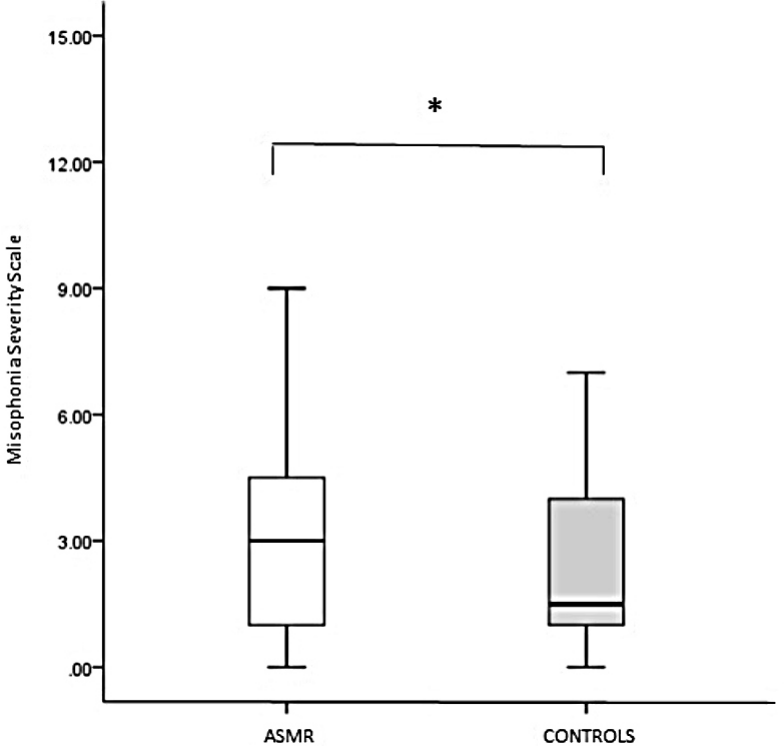
Scores for ASMR-responders (*N* = 64) and controls (*N* = 68) on the Misophonia Severity Scale. Sample medians are shown by vertical lines. *p < 0.05.

Scores on the Misophonia Severity Scale were analysed using independent-samples Mann–Whitney *U* test. This analysis revealed a significant group difference (*U*(132) = 1,733.000, *z* =  − 2.055, *p* = .040) due to ASMR-responders scoring higher (*Mdn* = 3.00) than controls (*Mdn* = 1.500) (Fig. 2). Additionally, 12.5% of ASMR-responders compared to 6% of the control sample reported 7 or above on the Misophonia Severity Scale which is treated as clinically significant misophonia level. However, this difference in severity levels between the groups was statistically non-significant [*χ*^2(1,*N*=132)^ = 3.09, *p* = .078).

A correlational analysis was also performed to examine the relationship between the Misophonia Severity Scale and the individual questions on the Misophonia Symptom Scale and the Misophonia Emotions and Behaviors Scale. This revealed significant positive correlations (all at *p* < .001) between all individual items and severity scores (Table 1), suggesting that severity of misophonia is related to increased scores on all items included in the Misophonia Symptom Scale and the Misophonia Emotions and Behaviors Scale.

**Table 1:** Correlation coefficients between individual questions on the Misophonia Symptom Scale and the Misophonia Severity Scale and between the Misophonia Emotions & Behaviors Scale and the Misopphonia Severity Scale.

Misophonia Symptom Scale	Misophonia Severity Scale
Q1	.421^***^
Q2	.364^***^
Q3	.370^***^
Q4	.418^***^
Q5	.311^***^
Q6	.458^***^
Q7	343^***^
**Misophonia Emotions and Behaviors Scale**	
Q1	429^***^
Q2	.456^***^
Q3	413^***^
Q4	484^***^
Q5	423^***^
Q6	.422^***^
Q7	308^***^
Q8	.334^***^
Q9	370^***^
Q10	.386^***^

**Notes.**

*** *p* < .001.

### Analysis based on female participants only

As there were more females in the control group a control analysis was also conducted in order to establish whether gender impacted the results. This entailed running the same analysis on female participants only. The two groups did not differ in terms of age [*t*(90) = 1.360, *p* = .177]. Performance on the MQ Total was analysed using an independent samples *t*-test which revealed a significant group difference [*t*(90) = 3.823, *p* =  < .001, *Cohen*′*sd* = 0.80) with ASMR-responders scoring higher (*M* = 26.22, *SD* = 10.30) than controls (*M* = 18.13, *SD* = 9.87). Subsequently, a 2 (group: controls, ASMR-responders) × 2 (subscales: MSS, MEBS) ANOVA revealed no interaction (*F*(1, 90) = 3.652, *p* = .059, ŋ*p*^2^ = .001) and a main effect of group [*F*(1, 90) = 14.615, *p* =  < .001, ŋ*p*^2^ = .140] with ASMR-responders scoring overall higher on both subscales (*M* = 13.11, *SE* = .80) compared to controls (*M* = 9.06, *SE* = .70). As with the primary analyses described above, performance on the Misophonia Severity Scale was analysed using an independent-samples Mann–Whitney *U* test. This analysis revealed a significant group difference (*U*(92) = 768.000, *z* =  − 2.178, *p* = .029) due to ASMR-responders scoring higher (*Mdn* = 4.00) than controls (*Mdn* = 3.00). Thus, qualitatively similar results were found when analysing female participants only to the analysis performed on both male and female participants.

There were, however, differences in the proportion of female participants who scored 14 and above on the Misophonia Symptom Scale compared to the proportion reported by groups including both male and female participants. Specifically, 35% of ASMR-responders compared to 26% non-responders scored 14 and above on this scale. However, similar to the primary findings involving data from males and females, this difference in proportions of participants scoring in the clinically relevant range was non-significant [*χ*^2(1,*N*=92)^ = 0.69, *p* = .40). Additionally, while the percentage of participants who reported 7 or above on the Misophonia Severity Scale was exactly the same as for the entire ASMR group (12.5%) only 3.8% of the control sample scored 7 or above on this scale. This result was also statistically non-significant [*χ*^2(1,*N*=92)^ = 2.40, *p* = .12).

In addition to these primary analyses, we also conducted analyses on a subset of control participants who reported ASMR (i.e., those who reported ASMR but were not recruited from social media sites for the experience). We observed a qualitatively similar pattern of results (see Supplemental Information 2).

## Discussion

The aim of this study was to examine whether ASMR is linked to increased levels of misophonia. The suggestion that these two phenomena may be linked was originally put forward by Barratt & Davis (2015) who pointed out that misophonia and ASMR might represent polar opposites of the same spectrum of sound sensitivity where human generated sounds elicit pleasurable tingling sensation in case of ASMR and negative physical and emotional responses in case of misophonia. In addition, based on previous findings suggesting that large proportion of ASMR-responders reported a negative reaction to “eating sounds” (Janik McErlean & Banissy, 2017), which are common misophonia inducers (Wu et al., 2014; Zhou, Wu & Storch, 2017), it was hypothesised that ASMR may be associated with heightened misophonia symptoms.

In order to establish whether ASMR is linked with elevated levels of misophonia a group of ASMR-responders and controls were administered the online Misophonia Questionnaire (Wu et al., 2014). The results showed that ASMR-Responders scored higher on the Misophonia Symptom Scale (MSS), which examines whether a person experiences sensitivity to sounds such as e.g., people eating or repetitive tapping. This suggests that ASMR is linked to enhanced sound sensitivity relative to general population. In addition, 36% of ASMR-Responders compared to 22% of non-responders scored 14 and above on this subscale of the Misophonia Questionnaire, indicating that on average they scored greater than or equal to a score of 2 (sometimes) for all of the symptom questions and thus a larger proportion of ASMR-Responders meet the criteria for misophonia diagnosis relatively to the control sample. These results are more conservative than previously reported (43%—Barratt, Spence & Davis, 2017), which most likely reflects methodological differences employed in the two studies. At the same time, the prevalence rate of misophonia in the general population reported in this study is similar to the previously reported rates among university students (20%—-Wu et al., 2014; 23.1%—Zhou, Wu & Storch, 2017). However, it is of note that the proportions of ASMR-responders and controls who meet the criteria for misophonia diagnosis based on cut-off scores on this scale are not statistically different.

ASMR-Responders also scored higher on the Misophonia Emotions and Behaviors Scale, which measures emotional and behavioural responses to certain sounds that include actively avoiding certain situations in anticipation of the sound, having violent thoughts or becoming physically aggressive. This suggests that heightened sound sensitivity present in ASMR as indicated by elevated scores on the Misophonia Symptom Scale in this group is associated with more severe negative behavioural and emotional outcomes relatively to control participants. In addition, ASMR-responders had increased scores on the Misophonia Questionnaire Total (MQ Total) scale, which is calculated by adding the two individual subscales (MSS and MEBS) together.

Further, ASMR-responders had increased scores on the Misophonia Severity Scale, which suggests greater level of severity of sound sensitivity in this population compared to controls. 12.5% of ASMR-responders compared to 6% of the control sample reported 7 or above on this scale, which is interpreted as clinically significant misophonia symptoms that can interfere with normal functioning. Although twice as many ASMR-responders reported clinically significant misophonia symptoms relative to control participants in this study, this difference was statistically non-significant and overall, these percentages are much lower than previously reported prevalence rates in the general population which is quite perplexing (19.9%—Wu et al., 2014; 16.6%—Zhou, Wu & Storch, 2017). This may suggest that misophonia experienced by the participants in this study is rarely bothersome to a point where it interferes with daily activities. Replicating current findings with another group of participants and perhaps using a different misophonia instrument will be an important next step, which will further verify current findings and shed more light on the relationship between the two conditions.

A large proportion of participants recruited outside of the ASMR Facebook site (47%) also indicated to experience ASMR based on the given description of the phenomenon. These participants were not included in the primary analysis as it was not possible to firmly exclude the possibility of them providing false positive answers as no additional questions regarding ASMR were asked. Nevertheless, it is possible that these individuals also genuinely experience this phenomenon. In order to establish whether this group (ASMR Non-Social Media) showed a similar pattern of results to ASMR Social-Media recruited from the aforementioned Facebook site dedicated to ASMR an additional analysis was conducted (see Supplemental Information 2). As the three groups had substantially more female than male participants, and gender did affect some of the results in this extra analysis, only data contributed by female participants is discussed. Current findings suggest that ASMR Non-Social Media scored significantly higher on MQ Total and overall on MSS and MEBS compared to ASMR Social-Media and controls. However, the difference between ASMR Social-Media and ASMR Non-Social Media was only marginally significant suggesting similar pattern of results across these two groups on overall scores. Results pertaining to the prevalence of clinically significant symptoms based on the ratings on the MSS showed ASMR Non-Social Media group to have a significantly higher proportion of individuals reporting clinically relevant symptoms compared to both controls and ASMR Social-Media. With respect to the reported severity levels there was no difference between ASMR Non-Social Media and ASMR Social-Media. There was also no statistically significant difference in prevalence rates in terms of the reported severity levels between ASMR Non-Social Media and ASMR Social-Media. Therefore, in general it appears that ASMR Non-Social Media and ASMR Social-Media show a similar pattern of results with respect to MQ Total, MSeverity and prevalence rates based on severity scores. They do, however, have a much higher prevalence rate (70.8%) of clinically relevant symptoms based on MSS scores compared to ASMR Social-Media (35%) and controls (26%), which was found to be statistically significant.

Therefore, similarly to participants recruited through ASMR Facebook site individuals recruited in a more systematic way who self-identified as experiencing ASMR also show increased levels of misophonia compared to controls. Moreover, their misophonia appears to be even more pronounced compared to ASMR-Responders recruited through ASMR community sites as evidenced by increased scores on all variables and a much larger proportion of them scoring within the clinically significant range on the MSS.

As the overall pattern of results with respect to misophonia was similar between ASMR Social-Media and ASMR Non-Social Media it appears that the latter group may have consistent experiences to those reporting ASMR from the social media community. However, in order to eliminate the possibility of a third variable accounting for this result (e.g., comorbidity with other conditions) a more thorough investigation controlling for such potentially confounding factors will need to be carried out in the future. The findings also suggest that when recruiting participants in a more systematic way (university students and acquaintances rather than special interests groups) almost half of the sample reported ASMR experiences, suggesting that this phenomenon may be highly prevalent in the general population. This could account for the huge popularity of the YouTube channels producing ASMR content. However, as mentioned earlier, stricter verification methods will need to be employed in the future in order to assess this claim.

Current results may also suggest that while individuals who experience ASMR may have increased overall levels of misophonia compared to controls, those who seek membership in online communities dedicated to ASMR may have lower levels of misophonia compared to individuals who experience ASMR but are either not aware of the phenomenon or do not actively engage in such online communities. It is feasible that only those individuals with moderate levels of misophonia would seek ASMR videos as they often include sounds which could also trigger misophonia in some ASMR-responders. However, as no questions regarding viewing habits were included in the questionnaire it is not possible to establish how many of ASMR Non-Social Media group were previously aware of this phenomenon and whether they engage in watching ASMR videos. The latter was also not established in case of the ASMR Social-Media group either. This shortcoming will need to be addressed in future research to gain better understanding of the obtained results.

Taken together, the findings reported here of heightened misophonia symptomatology among individuals who report ASMR relative to controls are in line with previous suggestions of a potential link between the two conditions (Barratt & Davis, 2015; Janik McErlean & Banissy, 2017; Fredborg, Clark & Smith, 2017; Rouw & Erfanian, 2017; Barratt, Spence & Davis, 2017). This further supports the notion that these two phenomena may indeed represent two ends of the same sound sensitivity spectrum and that they can co-occur in the same individuals. They also further suggest that there is a wide heterogeneity among ASMR-responders in terms of the triggers, which in some may produce positive while in others may lead to negative physiological, emotional and behavioural outcomes. In addition, both phenomena have been linked with a strong physical response to triggers. Misophonia has been associated with increased autonomic activation indexed with heart rate and galvanic skin response (Kumar et al., 2017). At the same time, pleasurable tingling sensation reported by ASMR-responders is the essence of ASMR experience (Barratt & Davis, 2015). Interestingly, pleasurable reaction to music experienced as ‘chills’ has also been linked to increased heart rate, electromyogram activity and respiration depth (Blood & Zatorre, 2001). Therefore, phenomenologically ASMR and misophonia appear similar, however, objective measures need to be employed in the future to provide physiological evidence for the sensory experience reported by ASMR-responders.

These findings are also interesting in light of the suggestion that ASMR and misophonia appear to rely on similar inducer-concurrent mechanisms as synaesthesia (Barratt & Davis, 2015). Indeed, the prevalence rate of synaesthesia among ASMR-responders appears to be greater compared to non-responders (Barratt & Davis, 2015), although more strict verification methods need to be implemented to verify this claim. In addition, all three phenomena have been associated with abnormal brain connectivity (Kumar et al., 2017; Smith, Fredborg & Kornelsen, 2016; Dovern et al., 2012), potentially suggesting shared mechanisms between misophonia, ASMR and synaesthesia. Specifically, compared to controls ASMR-responders at rest have been found to have less functional connectivity within the default mode network (DMN), increased connectivity between different sensory regions and reduced connectivity between the frontal lobes and regions involved in sensory and attentional processes (Smith, Fredborg & Kornelsen, 2016). These findings have been suggested to reflect diminished suppression of the multimodal experiences, which constitute the essence of ASMR. At the same time major theories postulate that synaesthesia arises as a result of reduced inhibition from multisensory (Grossenbacher & Lovelace, 2001) and executive control brain areas (Cohen Kadosh et al., 2009) to sensory regions. Misophonia has also been linked to abnormal functional connectivity. Specifically, it has been associated with increased activity in the anterior insular cortex (AIC), which has been linked to interoceptive ability and emotion processing (Critchley & Harrison, 2013) and enhanced functional connectivity between AIC and areas within the DMN suggesting that misophonia entails enhanced focus on internal signals coupled with attribution of exaggerated salience to otherwise ordinary sounds (Kumar et al., 2017). As no studies to date examined the neural activity underlying ASMR sensation while it is being experienced it is not clear whether similar results to those obtained during misophonic episodes would also pertain to this condition. It will be an important avenue to pursue in order to further examine the links between the two conditions.

Current results are also interesting in light of the reports of an association between misophonia, obsessive-compulsive disorder, anxiety and depressive symptoms (Wu et al., 2014; Zhou, Wu & Storch, 2017) and findings suggesting potentially heightened levels of psychological distress and increased levels of neuroticism among ASMR-responders (Janik McErlean & Banissy, 2017; Fredborg, Clark & Smith, 2017). However, a more systematic examination of the relationship between ASMR, misophonia, OCD, anxiety and depression is needed. This would also allow to constrain the current results. Specifically, in light of the findings suggesting a general pattern of comorbidity between psychiatric/clinical disorders (Kessler et al., 2005; Kessler et al., 1994), controlling for other symptoms/conditions (e.g., sensory sensitivity, OCD, depression, trauma) would allow to establish whether obtained results reflect a specific relationship between ASMR and misophonia, or whether current results are a bi-product of potential relationships between ASMR and other symptoms/conditions.

Additionally, as the current study uses a binary classification with respect to ASMR, examining the degrees of relationship between ASMR and misophonia is limited. Employing rating scales measuring the intensity of ASMR would be an important next step as it would allow for more comprehensive examination of the relationship between ASMR and misophonia and other conditions which also exist on a continuum.

## Conclusions

In summary, the current findings suggest increased levels of misophonia among individuals reporting ASMR relative to controls with respect to all subscales of the Misphonia Questionnaire (Wu et al., 2014) including Misophonia Symptom Scale, Misophonia Emotions and Behaviors Scale and Misophonia Severity Scale. These results are in line with previous suggestions that ASMR and misophonia represent two ends of the same sound sensitivity spectrum (Barratt & Davis, 2015).

##  Supplemental Information

10.7717/peerj.5351/supp-1 Supplemental Information 1 Anonymous data Output containing answers to individual questions and computed final scores. This data has been cleaned and anonymised.

10.7717/peerj.5351/supp-2 Supplemental Information 2 Supplemental Results Results of supplemental analysis conducted on data contributed by individuals recruited outside of social media sites dedicated to ASMR who also reported to experience ASMR

10.7717/peerj.5351/supp-3 Supplemental Information 3 Questionnaire text

## Additional Information and Declarations

### Data Availability

The following information was supplied regarding data availability: The raw data are provided in a Supplemental File.
