# Supplementary material for: Increased misophonia in self-reported Autonomous Sensory Meridian Response
Source: PeerJ. 2018 Aug 6;6:e5351. doi: 10.7717/peerj.5351 (PMC6084287; doi:10.7717/peerj.5351)
Supplement: Supplemental Information 2 — Results of supplemental analysis conducted on data contributed by individuals recruited outside of social media sites dedicated to ASMR who also reported to experience ASMR [file peerj-06-5351-s002.docx]

***Analyses based on participants recruited outside Facebook ASMR Group***

As a large group of participants (47%) recruited outside of the ASMR Facebook site also reported to experience ASMR an additional analysis was run to establish whether these individuals would show a similar pattern of results with regards to the misophonia levels to ASMR Social-Media group recruited via the aforementioned site. The ASMR Non-Social Media group comprised of 60 participants (48 females, 12 males, age: M = 25.11, SD = 10.06, range = 18-63). The three groups (controls, ASMR Social-Media, ASMR Non-Social Media) did not significantly differ in terms of age [F (2,191) = 2.414, p = .092] or gender [*^  =  ^*= 5.476, p = .065).

Performance on the MQ Total was analysed using a One-Way ANOVA which revealed statistically significant differences between groups [F (2,191) = 29.88, p < .001, ŋp² = .24) with ASMR Non-Social Media scoring the highest (M = 31.51, SD = 12.24) followed by ASMR Social-Media (M = 24.09, SD = 10.48) and controls (M = 16.80, SD = 9.51). Bonferroni-corrected pairwise comparisons revealed statistically significant differences between the three groups (all at p < .001) with respect to MQ Total scores.

Subsequently, a 3 (group: controls, ASMR Social-Media, ASMR Non-Social Media) x 2 (subscales: MSS, MEBS) ANOVA was conducted to examine whether this result was due to group differences on either of the two subscales, namely the Misophonia Symptom Scale and Misophonia Emotions and Behaviors Scale. This revealed no interaction [F (2,189) = .922, p = .399, ŋp² = .010], and a main effect of group [F (2,189) = 29.88, p <.001, ŋp² = .240]. Bonferroni-corrected pairwise comparisons showed statistically significant differences (all at p < .001) on overall scores on both subscales between all three groups with ASMR Non-Social Media scoring the highest (M = 15.75, SE = .70) followed by AMR-responders-A (M = 12.05, SE = .62) and controls (M = 8.40, SE = .60) respectively. Additionally, 68.3% of ASMR Non-Social Media group reported clinically significant symptoms on the Misophonia Symptom Scale compared to 36% of ASMR Social-Media and 22 % controls. This difference in the prevalence of clinically significant symptoms of misophonia between the three groups was statistically significant [*^  = ^* = 29.26, p< .001, V = .39). As the chi square test between ASMR Social-Media and controls was previously non-significant two post hoc chi-square tests were conducted to compare the scores between ASMR Social-Media and ASMR Non-Social Media [*^  = ^* = 13.014, p< .001, V = .32] and between ASMR Non-Social Media and controls [*^  = ^* = 27.735, p< .001, V = .46], which revealed statistically significant results in both instances (both surviving multiple comparisons correction).

Scores on the Misophonia Severity Scale were analysed using Kruskal-Wallis test which also revealed statistically significant group differences [*^  = ^* = 12.997, p = .002) with ASMR Social-Media scoring (Mdn = 3.00), controls (Mdn = 1.500) and ASMR Non-Social Media (Mdn = 4.00). Post hoc pairwise comparisons using Mann Whitney U tests revealed that the only group difference which survived multiple comparisons correction was between controls and ASMR Non-Social Media [U (128) = 315.00, z = -3.513, p < .001] while the difference between controls and ASMR Social-Media did not survive the correction [U (134) = 1733.00, z = - 2.055, p = .040, uncorrected value] and the difference between ASMR Non-Social Media and ASMR Social-Media was non-significant [U (124) = 1585.50, z = -1.695, p = .090, uncorrected value]. In addition, 20% of ASMR Non-Social Media group reported clinically significant misophonia severity based on the cut-off score of 7 on the Misophonia Severity scale compared to 12.5 % of ASMR Social-Media and 6% controls. However, chi-square test revealed marginally non-significant group differences [*^  = ^* = 5.80, p = .054).

As the ASMR Non-Social Media group also contained more females than males additional control analyses were run based on the data contributed only by female participants from the three groups: ASMR Social-Media (N = 40, age: M = 29.02, SD = 6.3, range: 18-38), controls (N = 52, age: M = 26.38, SD = 10.96, range: 18-64) and ASMR Non-Social Media (N = 48, age: M = 23.46, SD = 7.98, range: 18 – 63). As there were group differences in terms of age [F (2, 139) = 4.373, p = .014, ŋp² = .06] a correlational analysis was performed to establish whether age correlates with any of the study variables which revealed no statistically significant correlations i) Pearson’s correlations between age and MSS: r = .07, p = .383; age and MEBS: r = .03, p = .666; age and MQ Total: r = .02, p = .823; ii) Spearman’s correlation between age and MSeverity: r_s_ = .127 p = .135. Based on these results age was not included as a covariate in subsequent analysis.

Performance on the MQ Total was analysed using a One-Way ANOVA which revealed statistically significant differences between groups [F (2,139) = 20.436, p < .001, ŋp² =.18) with ASMR Non-Social Media scoring highest (M = 31.83, SD = 12.03) followed by ASMR Social-Media (M = 26.22, SD = 10.30), and controls (M = 18.13, SD = 9.87). Bonferroni-corrected pairwise comparisons revealed statistically significant differences between ASMR Social-Media and controls (p < .001), controls and ASMR Non-Social Media (p< .001) and a marginally significant difference between ASMR Social-Media and ASMR Non-Social Media (p =. 049).

Subsequently, a 3 (group: controls, ASMR Social-Media, ASMR Non-Social Media) x 2 (subscales: MSS, MEBS) ANOVA was conducted to examine whether this result was due to group differences on either of the two subscales, namely the Misophonia Symptom Scale and Misophonia Emotions and Behaviors Scale. This revealed a marginally significant interaction [F (2,137) = 3.087, p = .049, ŋp² = .043], and a main effect of group [F (2,137) = 20.436, p <.001, ŋp² = .230]. Bonferroni-corrected pairwise comparisons showed statistically significant differences between ASMR Social-Media and controls (p < .001), controls and ASMR Non-Social Media (p< .001) and a marginally significant difference between ASMR Social-Media and ASMR Non-Social Media (p =. 049) with ASMR Non-Social Media scoring the highest (M = 15.91, SE = .78), followed by AMR-responders-A (M = 13.11, SE = .85) and controls (M = 9.07, SE = .74). Follow-up independent samples t-tests which survived multiple comparisons correction revealed statistically significant group differences between ASMR Social-Media [M = 13.70, SD = 7.40] and controls [M = 8.25, SD = 5.33] on MEBS [t (68.090) = 3.936, p <.001, Cohen’s d = .84 ]; between ASMR Social-Media [M = 12.52, SD = 5.83] and ASMR Non-Social Media [ M = 17.10, SD = 6.27] on MSS [t(86) = -3.516, p <.001, Cohen’s d = .75]; between ASMR Non-Social Media [M = 17.10, SD = 6.27] and controls [ M = 9.88, SD = 6.00] on MSS [ t(98) = -5.878, p <.001, Cohen’s d = 1.17]; and between ASMR Non-Social Media [ M = 14.72, SD = 7.55] and controls [M = 8.25, SD = 5.33] on MEBS [t(83.887) = -4.919, p <.001, Cohen’s d = .99].

70.8% of female only ASMR Non-Social Media reported clinically significant scores on the Misophonia Symptom Scale compared to 35% of ASMR Social-Media and 26% controls. This difference in the prevalence of clinically significant symptoms between the three groups was statistically significant [*^  = ^* = 21.46, p< .001, V = .39). As the chi square test between ASMR Social-Media and controls was previously found to be non-significant two post hoc chi-square tests were conducted to compare the scores between ASMR Social-Media and ASMR Non-Social Media [*^  = ^* = 11.30, p< .001, V = .35] and between ASMR Non-Social Media and controls [*^  = ^* = 19.28, p< .001, V = .44], which revealed statistically significant results in both instances (both surviving multiple comparisons correction).

Scores on the Misophonia Severity Scale were analysed using Kruskal-Wallis test, which revealed statistically significant group differences [*^  = ^* = 10.661, p = .005) with ASMR Social-Media scoring (Mdn = 4.00), controls (Mdn = 3.00) and ASMR Non-Social Media (Mdn = 4.00). Post hoc pairwise comparisons using Mann Whitney U tests revealed a non-significant group difference between ASMR Social-Media and ASMR Non-Social Media [U(88) = 834, z = 1654.00, p = .285 uncorrected value]; a non-significant group difference between controls and ASMR Social-Media as it did not survive multiple comparisons correction [U(92) = 768.00, z = -2.178, p = .029 uncorrected value]; and a statistically significant difference between controls and ASMR Non-Social Media [U(88) = 805.500, z = -3.091, p = .002, uncorrected value].

25% of ASMR Non-Social Media reported clinically significant misophonia severity based on the cut-off score of 7 on the MSeverity scale compared to 12.5% ASMR Social-Media and 3.8% controls, which was a statistically significant group difference [*^  = ^* = 9.57, p = .008, V= .26). As the chi square test between ASMR Social-Media and controls was previously non-significant two post hoc chi-square tests were conducted to compare the scores between ASMR Social-Media and ASMR Non-Social Media [*^  = ^* = 2.18, p=.139, V = .15] which revealed a non-significant result and between ASMR Non-Social Media and controls [*^  = ^* = 9.27, p< .002, V = .30] which was statistically significant and survived multiple comparison correction.
