# Supplementary material for: Increased misophonia in self-reported Autonomous Sensory Meridian Response
Source: PeerJ. 2018 Aug 6;6:e5351. doi: 10.7717/peerj.5351 (PMC6084287; doi:10.7717/peerj.5351)
Supplement: Supplemental Information 3 [file peerj-06-5351-s003.docx]

Appendix 2. Questionnaire

1. Demographic section

What is your gender? [Female/Male/Other]

What is your age? _____

ASMR can be defined as a pleasurable tingling sensation which originates on scalp and spreads down the spine and through the whole body and which is typically induced by certain sounds (e.g. turning pages, crinkly wrapping paper, finger tapping), watching someone perform repetitive mundane actions (e.g. folding towels, going through items in a handbag), watching someone closely inspecting day-to-day objects, hearing whisper, watching someone's hair being brushed or watching videos with various role plays (visit to a doctor, spa or a shop).

Do you experience ASMR? [Yes/No/Other]

1. Misophonia section

ASMR is often induced by tapping, chewing, whispering etc. but for some people some of these sounds might be unpleasant and distressing. This questionnaire aims to establish if people who experience ASMR perceive some of these sounds as aversive. These sounds may lead to negative emotions, irritation and anger. Please note that the word ‘sensitive’ in the questions below refers to the negative emotions and irritation induced by certain sounds rather than positive and pleasant sensitivity experienced during ASMR.

Misophonia Questionnaire (as per Wu, Lewin, Murphy, & Storch, 2014)

Please rate how much the following statements describe you on a scale from 0 to 4, 0 being “Not at all true” and 4 being “Always true.”

In comparison to other people, I am sensitive to the sound of:

1. People eating (e.g. chewing, swallowing, lips smacking, slurping, etc.). [Not at all True (0) to Always True (4)]

2. Repetitive tapping (e.g. pen on table, foot on floor, etc.). [Not at all True (0) to Always True (4)]

3. Rustling (e.g. plastic, paper, etc.). [Not at all True (0) to Always True (4)]

4. People making nasal sounds (e.g. inhale, exhale, sniffing, etc.). [Not at all True (0) to Always True (4)]

5. People making throat sounds (e.g. throat-clearing, coughing, etc.). [Not at all True (0) to Always True (4)]

6. Certain consonants and/or vowels (e.g. “k” sounds, etc.). [Not at all True (0) to Always True (4)]

7. Environmental sounds (e.g. clock ticking, refrigerator humming, etc.). [Not at all True (0) to Always True (4)]

8. Other: ______________________________ [Not at all True (0) to Always True (4)]

Now rate how often the subsequent statements occur, 0 being “Never” and 4 being “Always.”

Once you are aware of the sound(s), because of the sound(s), how often do you:

1. Leave the environment to a place where the sound(s) cannot be heard anymore? [Never (0) to Always (4)]

2. Actively avoid certain situations, places, things, and/or people in anticipation of the sound(s)? [Never (0) to Always (4)]

3. Cover your ears? [Never (0) to Always (4)]

4. Become anxious or distressed? [Never (0) to Always (4)]

5. Become sad or depressed? [Never (0) to Always (4)]

6. Become annoyed? [Never (0) to Always (4)]

7. Have violent thoughts? [Never (0) to Always (4)]

8. Become angry? [Never (0) to Always (4)]

9. Become physically aggressive? [Never (0) to Always (4)]

10. Become verbally aggressive? [Never (0) to Always (4)]

11. Other: ______________________________ [Never (0) to Always (4)]

Please rate the severity of your sound sensitivity on the following scale from 1 (minimal) to 15 (very severe) by choosing one number that best describes your experiences. Again it relates to the negative sensitivity and distressing sounds only. Please consider the number of sounds that you are sensitive to, the degree of distress, and the impairment in your life due to your sound sensitivities. Please note that some numbers don’t have anything written next to them. They just represent a transition in severity from 1 to 15 and the scale is designed this way to give you more flexibility when rating the severity of your sound sensitivity. So for instance 2 is more severe than 1, 5 is more severe than 4, 6 more severe than 5 and so on until 15 which represents the highest severity. If you do not have any sound sensitivities, please check 0.

1. I have no sound sensitivity.
2. Minimal within range of normal or very mild sound sensitivities. I spend little time resisting or being affected by my sound sensitivities. Almost no or no interference in daily activity.
3. Mild sound sensitivities. Mild sound sensitivities that are noticeable to me and to an observer, cause mild interference in my life and which I may resist or be affected for a minimal period of time. Easily tolerated by others.

5.

6.

7. Moderate sound sensitivities. Sounds sensitivities that cause significant interference in my life and which I spend a great deal of conscious energy resisting or being affected by. Require some help from others to function in daily activity.

8.

9.

10. Severe sound sensitivities. Sound sensitivities that are crippling to me, interfering so that daily activity is “an active struggle.” I may spend full time resisting my sound sensitivities or being affected by them. Require much help from others to function

11.

12.

13. Very severe sound sensitivities. Sound sensitivities that completely cripple me so that I require close supervision over eating, sleeping, and so forth. It is hard to function on a day-to-day basis because of this

14

15.
